# Allostatic Load, Cigarette Smoking, and Lung Cancer Risk

**DOI:** 10.3390/cancers16183235

**Published:** 2024-09-23

**Authors:** Yufan Guan, Jie Shen, Kai Zhang, Bernard F. Fuemmeler, Hua Zhao

**Affiliations:** 1 Department of Public Health Sciences, School of Medicine, University of Virginia, Charlottesville, VA 22903, USA; 2 Department of Environmental Health Sciences, School of Public Health, State University of New York at Albany, Albany, NY 12222, USA; 3 Department of Family Medicine, School of Medicine, Virginia Commonwealth University, Richmond, VA 23284, USA

**Keywords:** allostatic load, lung cancer, cigarette smoking

## Abstract

**Simple Summary:**

Allostatic load is a biomarker of chronic stress. It has been implicated in the etiology of multiple chronic diseases. However, the role of allostatic load in lung carcinogenesis is still largely unknown. Given cigarette smoking could modify levels of allostatic load, the investigation of the relationship between allostatic load and lung cancer risk is of particular interest. This work thus aims to carry out the first study to prospectively assess the association.

**Abstract:**

**Background:** Allostatic load (AL) is a biomarker of chronic stress associated with various chronic diseases. No study has evaluated the relationship between AL and lung cancer risk. **Methods:** To address this gap, we analyzed the association between AL and the development of lung cancer in 344,380 participants from the UK Biobank. **Results:** During the follow-up period from 2006 to 2020, 2517 participants were diagnosed with incident lung cancer. Participants who developed lung cancer had significantly higher AL compared to cancer-free controls (mean: 3.49 vs. 2.87, *p* < 0.001). In the multivariate analysis, a marginally significant association was observed between higher AL and increased lung cancer risk (per one AL unit: Hazard Ratio [HR] = 1.02, 95% Confidence Interval [CI]: 0.99, 1.04). In the categorical analysis, individuals with high AL (AL > 2) had a 15% higher risk of lung cancer compared to those with low AL (AL ≤ 2) (HR = 1.15, 95% CI: 1.05, 1.25). Stratified analyses revealed that this increased risk was only observed in former (HR = 1.38, 95% CI: 1.06, 1.43) and current smokers (HR = 1.25, 95% CI: 1.10, 1.42) but not in never-smokers (HR = 0.93, 95% CI: 0.74, 1.17). Moreover, we found that demographics, socioeconomics, and other health behaviors could modify the risk association. Finally, among cigarette smoking-related variables, a significant trend of increasing AL was observed with higher pack-years, longer smoking duration, earlier age of smoking initiation, and later age of smoking cessation. **Conclusions:** These findings suggest that higher AL is associated with an increased risk of lung cancer. The results need to be further confirmed in additional studies.

## 1. Introduction

Allostatic load (AL), a biomarker of chronic stress, reflects the physiological “wear and tear” resulting from an individual’s exposure to stressors over their lifetime [1]. High AL scores have been significantly associated with worsened psychiatric symptoms, cognitive decline, physical deterioration, and increased risk of a variety of chronic diseases and mortalities [2]. Recent research has also explored AL’s role in cancer outcomes, mainly in breast cancer [3]. Elevated AL scores are linked to increased risk [4], poorer tumor characteristics [5], and mortality [6,7] in breast cancer. So far, only one study has ever been done on lung cancer. In non-small cell lung cancer patients, elevated AL was found to correlate with worse overall mortality [8].

In fact, lung cancer, the top cause of cancer-related death in the US and the second most prevalent cancer among both men and women, is of relevance to AL. Several lines of evidence support AL’s role in lung cancer etiology. First, past studies have demonstrated that cigarette smoking, a major risk factor for lung cancer, is associated with increased AL [9,10,11,12]. Our analysis of SWAN data revealed that both current and former smokers exhibit significantly higher AL levels compared to never-smokers [13]. Second, stressful life events have been linked to a higher risk of lung cancer [14,15,16,17]. Third, recent research indicates that stress contributes to lung carcinogenesis [18,19]. Chronic stress has been shown to facilitate lung tumorigenesis by promoting exocytosis of IGF2 in lung epithelial cells and increasing metastasis through neutrophil-mediated changes in the tumor microenvironment [19]. Fourth, individuals with higher levels of stress are more likely to engage in heavy smoking and are less likely to successfully quit in comparison to their counterparts [20,21]. Despite the compelling evidence above, no epidemiological studies have yet assessed the relationship between AL and lung cancer risk.

In this study, we aim to address this gap by using the valuable resources of the UK Biobank to assess whether AL is associated with an increased risk of lung cancer. We also analyzed smoking-related variables to clarify their relationship with AL.

## 2. Methods

Study Cohort: The UK Biobank project, conducted from 2006 to 2010, enrolled over 500,000 volunteers aged 46–69 across the UK [22]. Participants completed a detailed touch-screen questionnaire, provided biological samples, and underwent various physical measurements. For our study, we identified 502,241 participants as the initial cohort. We focused on individuals without a history of cancer at the time of enrollment, excluding those with benign tumors, carcinoma in situ, nonmelanoma skin cancer, or cancers of unknown prevalence. Additionally, participants lacking complete information on any of the 11 factors required to construct AL scores were excluded. This stringent selection process resulted in a final analysis cohort of 344,380 participants.

Lung Cancer Cases Ascertainment: For this study, incident lung cancer cases were identified using ICD-10 codes reported in the UK Biobank database (Supplement Table S1). A stepwise selection of study participants was shown in Supplement Figure S1. Participants were followed up until 31 December 2020, with a median follow-up period of 11.6 years. Incident lung cancer cases were defined as those diagnosed with malignant neoplasms of the lung during the follow-up period. We excluded participants diagnosed with lung cancer within one year of their initial enrollment (N = 208) to ensure robust data. From the entire study population of 344,380 participants, we identified 2517 individuals with incident lung cancer.

AL Score Construction: In this study, we developed an Allostatic Load (AL) score utilizing eleven factors derived from baseline measurements (Supplement Table S2). Following the methodology outlined by Zhao and Chyu et al. [4,13,23], the AL score included biomarkers across three health domains: cardiovascular (Systolic Blood Pressure [SBP], Diastolic Blood Pressure [DBP], and Pulse Rate [PR]), inflammatory (C-reactive Protein [CRP]), and metabolic indicators (High-Density Lipoprotein [HDL], Waist-to-Hip Ratio, Abnormal Cholesterol, Triglycerides [TG], Hemoglobin A1c [HbA1c], and Creatinine). Each biomarker was assessed against established clinical thresholds, with scores of 0 or 1. Additionally, the history of medication use for metabolic diseases and hypertension was included as a factor, with a score of 1 given to those with such a history and 0 to those without. Consequently, the AL score ranged from 0 to 11, summing all factors. Based on the distribution, we further categorized the AL score into a dichotomized variable, either the low AL group (AL ≤ 2) or the high AL group (AL > 2).

Assessment of covariates: To adjust potential confounders, we included a list of covariates. They were age, gender, race, family history of lung cancer, education level, employment, family income, the Townsend deprivation index, cigarette smoking, alcohol consumption, sleep quality, and physical activity. For most categorical covariates (including gender, race/ethnicity, employment status, smoking status, alcohol consumption, and sleep quality), we used the UK Biobank codebook to define the category. Family history of lung cancer was determined by whether any first- or second-degree family member had been diagnosed with lung cancer. The income category was determined using £31,000 as the cutoff point. The Townsend deprivation index, derived from participants’ postcodes, was classified as low or high based on the median value. Education levels were categorized into “High school or less” and “College/professional”. Physical activity was evaluated using MET scores and classified into “Low”, “Moderate”, and “High”.

Statistical analysis: First, we compared differences in the mean (for continuous variables) and distribution (for categorical variables) of each covariate between lung cancer cases and cancer-free controls. Next, we compared the distribution of AL scores between these groups, treating AL as a continuous and categorical variable. The Student’s *t*-test was utilized to identify differences between continuous variables, while the Chi-square test was applied to detect differences between categorical variables. Then, the Cox Proportional Hazard regression model was employed to assess the association between the AL score and lung cancer risk. The event of interest was the first diagnosis of lung cancer. For lung cancer cases, follow-up time was defined from baseline to the date of lung cancer diagnosis, and for individuals who developed other cancers, the follow-up period extended from baseline to the date of diagnosis of these other cancers, with censoring at the time of diagnosis. For participants lost to follow-up, the period was from baseline to the date of the last follow-up. Additionally, Kaplan–Meier survival curves with the log-rank test were used to examine differences in lung cancer risk between different AL groups. To refine our analysis, we further conducted multivariate Cox Proportional Hazard regression models, adjusting for demographics (age, gender, and race), family history of lung cancer, socioeconomic status (education level, employment, and family income, and the Townsend deprivation index), and lifestyle factors (cigarette smoking, alcohol consumption, sleep quality, and physical activity). We included pack-years in the model to adjust the impact of cigarette smoking amount further. The proportional hazards assumption was tested, and a non-proportional hazards model was used if violated. Model fitting was assessed using the Likelihood Ratio Test. Subsequently, we conducted a stratified analysis to explore the association between AL and lung cancer risk factors that differed by covariates. Potential statistical interactions were noted. Finally, ANOVA was used to examine the relationship between smoking-related variables (pack-years, age of smoking initiation, age of smoking cessation, and years of smoking) and AL. Trend analyses assessed the dose–response relationship between smoking-related variables and AL scores. All statistical tests were two-sided, with *p*-values below 0.05 deemed statistically significant. The studies were performed using R version 4.3.0.

## 3. Results

Three hundred forty-four thousand three hundred eighty participants were included in the final analysis, with a median follow-up of 11.6 years. During this period, 2517 incident lung cancer cases were recorded, with a median follow-up time of 7.0 years for these cases. The age-standardized incidence rate for males was 80.65 per 10,000 population, while for females, it was 78.03 per 10,000 population. Table 1 outlines the distribution of selected characteristics between lung cancer cases and the cancer-free controls. Compared to controls, lung cancer cases were older (61.6 vs. 56.2 years, *p* < 0.001) and more likely to be male (52.05% vs. 47.13%, *p* < 0.001), White (96.82% vs. 94.37%, *p* < 0.001), and have a family history of lung cancer (13.91% vs. 7.71%, *p* < 0.001). For socioeconomic status-related variables, lung cancer cases were more likely to be retired (52.01% vs. 31.91%, *p* < 0.001), have low income (59.00% vs. 40.32%, *p* < 0.001), and live in the area with high levels of deprivation, as indicated by the Townsend deprivation score (63.61% vs. 49.44%, *p* < 0.001). In terms of healthy behaviors, lung cancer cases were less likely to be never-smokers (13.35% vs. 55.11%, *p* < 0.01) and have quality sleep (19.67% vs. 24.51%, *p* < 0.001).

The distribution of AL scores in lung cancer cases and cancer-free controls is presented in Table 2. The AL scores in this study ranged from 0 to 11. However, only 6 participants had an AL score of 10 or higher. The most frequent AL score was 3, representing approximately 22.61% of the cases and 20.40% of the controls. Overall, the AL distribution in the case group was left-skewed, with only about 11.36% of participants having an AL score of 6 or more. A significant difference was observed when comparing AL distribution between cases and controls (*p* < 0.001). Compared to cancer-free controls, lung cancer cases were more likely to be in the high AL categories (AL: 3 to 9) than the low AL categories (AL: 0 to 2). The mean AL score for lung cancer cases was 3.49, significantly higher than the mean score of 2.87 for the non-cases population (*p* < 0.001). Based on AL score distribution, we combined participants with AL scores of ≤2 into one category and AL scores of >2 into another category, creating a dichotomized variable, AL Category. A significant difference in the distribution of AL Category (high vs. low) was observed between lung cancer cases and cancer-free controls (*p* < 0.001), with lung cancer cases more likely to be in the high AL group than non-cases (71.75% vs. 55.51%, *p* < 0.01).

**Figure 1:**
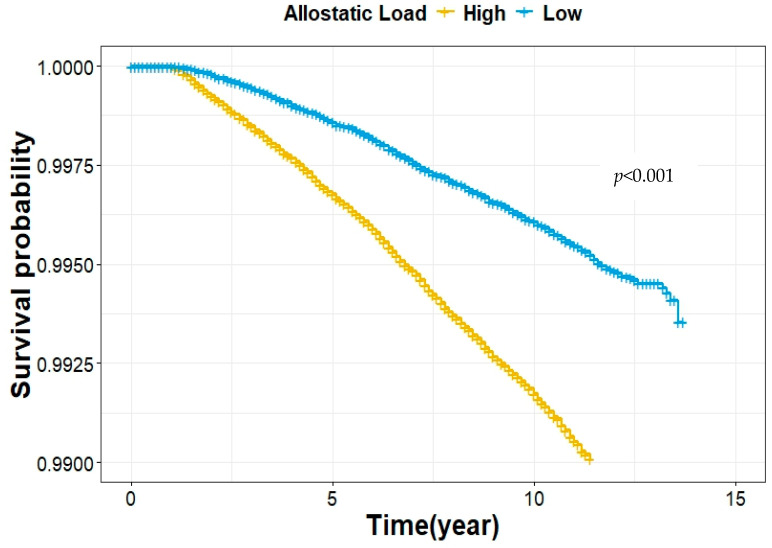
Kaplan–Meier survival estimates for the association between the AL score category and lung cancer risk.

Then, we investigated the association between AL and lung cancer risk, as detailed in Table 2. First, in the univariate Cox Proportional Hazard regression analysis, we found a 23% increase in lung cancer risk for each unit increase in the AL score (HR = 1.23, 95% CI: 1.20, 1.26) if AL was treated as a continuous variable. In the multivariate Cox regression analysis, demographic variables (age, gender, and race), family history of lung cancer, socioeconomic status (education, employment status, income, and Townsend deprivation index), and healthy behaviors (cigarette smoking, pack-years, alcohol consumption, sleep quality, and physical activity) were included in the model, and we observed a marginally significant association (HR = 1.02, 95% CI: 0.99, 1.04) between AL score and lung cancer risk. No significant violation of the Cox proportional hazards assumption for any model was confirmed using the Schoenfeld residuals test. Then, treated as a categorical variable, participants in the high AL group (AL > 2) had a 2.08-fold increased risk of lung cancer compared to those in the low AL group (AL ≤ 2) in the univariate analysis (HR = 2.08, 95%CI: 1.91, 2.27). Figure 1 illustrates the Kaplan–Meier survival curves, indicating that participants with high AL scores (AL > 2) had a significantly higher likelihood of developing lung cancer compared to those with low AL scores (AL ≤ 2) (*p* < 0.001). In further multivariate analysis, the risk associated with the high AL group remained (HR = 1.15, 95% CI: 1.05, 1.25).

Subsequently, we investigated whether the association between AL and lung cancer risk differed by covariates (Table 3). The risk associated with high AL was slightly higher among those who were younger (<57 years old), women, Whites, and had higher levels of socioeconomic status (>high school education, higher income, employed, retired, and low Townsend deprivation index) than their counterparts. No statistical interaction was observed. For cigarette smoking, the risk was only observed among former (HR = 1.38, 95% CI: 1.20, 1.59) and current smokers (HR = 1.17, 95% CI: 1.02, 1.35), and not among never-smokers (HR = 0.93, 95% CI: 0.74, 1.17). Similarly, the risk was only observed among those with moderate (HR = 1.24, 95% CI: 1.06, 1.46) and heavy alcohol consumption (HR = 1.25, 95% CI: 1.09, 1.44), and not among never/occasional drinkers (HR = 1.11, 95% CI: 0.93, 1.34). In addition, significant interactions were observed between cigarette smoking and alcohol drinking with the AL category (former and current vs. never-smokers: *p* = 0.001 and 0.031, respectively; heavy vs. never/occasional drinkers: *p* = 0.030). The risk also differed by physical activity and sleep quality. A significant association was only observed among those with moderate and high levels of physical activity, and never/rarely and sometimes insomnia, but not among those with low levels of physical activity and frequent insomnia.

**Figure 2:**
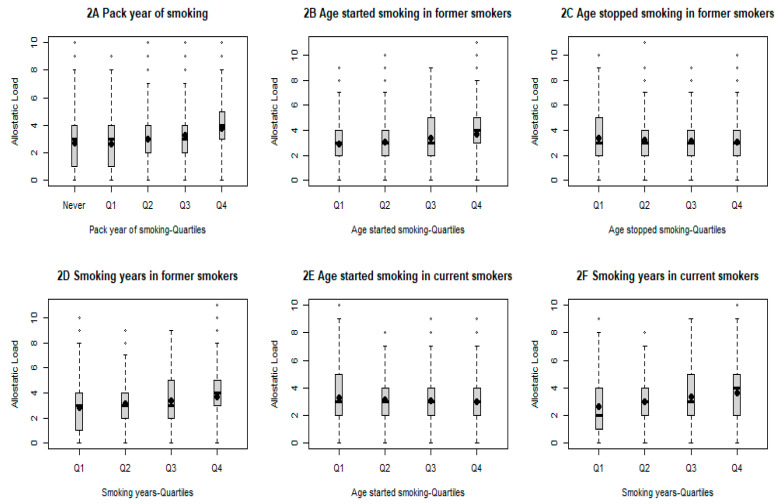
Comparison of AL score by selected smoking-related variables. All *p* for trend less than 0.001.

Our final analysis further investigated the association between various smoking-related factors and AL (Table 4). As expected, all analyzed smoking-related variables, including smoking status, pack-years of smoking among all study participants, age of smoking initiation, and years of smoking among former and current smokers, respectively, and age of smoking cessation among former smokers, were significantly associated with levels of AL (*p* < 0.001, respectively). Additionally, a significant trend of increasing AL was observed for changing smoking status from never or former to current (*p* < 0.001) and increasing pack-years from never to the highest quartile (*p* < 0.001) (Figure 2). A similar trend was also observed for increasing years of smoking among both former and current smokers and increasing age of smoking cessation among former smokers (*p* < 0.001, respectively). On the other hand, a significant trend of increasing AL was observed for increasing age of smoking initiation (*p* < 0.001).

## 4. Discussion

This is the first study to assess the relationship between AL and lung cancer risk in a cohort. In summary, we found that higher levels of AL are associated with a 15% increased risk of lung cancer after adjusting for demographic, socioeconomic, and health behavioral factors, as well as a family history of cancer. In stratified analysis, the significant risk association with AL was only observed among former and current smokers but not among non-smokers. We also found that demographics (age, gender, and race), socioeconomics (education, income, employment, and Townsend deprivation index), and other health behaviors (alcohol drinking, physical activity, and sleep quality) could modify the risk association. In a further analysis of the relationship between smoking-related variables and AL, significant trends of increasing AL were observed with the increase in pack-years, smoking years, and age of smoking cessation (former smokers only) and the decrease in age of smoking initiation.

A significant association between AL and lung cancer is expected—evidence from epidemiologic and laboratory studies supports the role of stress in the etiology of lung cancer. In a case–control study, Jafri et al. reported that lung cancer patients are significantly more likely to have had a major stressful life event within the preceding 5 years [14]. The use of β-blockers may be protective against lung cancer. In an earlier study, Levav et al. found that bereaved parents who had lost an adult son in an accident had a significantly higher chance of developing lung cancer later in life [24]. Furthermore, in a recent study in Chicago, exposure to increased neighborhood violence was found to be associated with an increased risk of lung cancer, possibly through activating physiological stress responses [25]. In laboratory mice, Jang et al. reported that chronic stress could accelerate lung tumorigenesis by promoting the exocytosis of IGF2 in lung epithelial cells [18]. Also, glucocorticoids released during chronic stress cause neutrophil extracellular trap (NET) formation, establish a metastasis-promoting microenvironment, and ultimately accelerate lung tumor metastasis [19].

Furthermore, Liu et al. reported that the combination of chronic stress and smoke could exacerbate the development of lung cancer in A/J mice [26]. Such a synergistic effect was also reported in a human study to show the combination of smoking and depression can contribute to decreases in NK cell activity, which serves as the first line of defense against tumors [27]. Our findings are consistent with those literature reports. In this study, we found significant statistical interactions between AL and smoking status. Specifically, the risk association with AL only exists among former and current smokers but not among non-smokers. In further analysis, we investigated whether the association between AL and lung cancer risk differed by the amount and duration of cigarette smoking. Among former smokers, there was a significant trend of increasing lung cancer risk with the increase in pack-years and years of smoking from lowest to highest quartile (both *p* for trend < 0.001). This further strengthens the notion that there is a synergistic effect between stress, represented by AL here, and cigarette smoking on lung cancer risk. In addition, we found a significant statistical interaction between alcohol consumption and AL in lung cancer risk. This is likely because heavy drinkers are more likely to be ever smokers in this study (*p* < 0.001).

One exciting but puzzling finding in this study is that the risk association is more evident among those with higher levels of socioeconomic status (SES) at both individual (education, employment, and income) and neighborhood levels (Townsend deprivation index). On the one hand, in our study population, individuals with lower levels of education and income, unemployment, and living in areas with high levels of Townsend deprivation index have statistically significantly higher AL levels than their counterparts. On the other hand, lower SES has been linked to worse lung cancer outcomes [28]. For example, in a pooled case–control study with 17,021 cases and 20,885 controls, an elevated risk between lung cancer and low SES was observed, even after the adjustment for cigarette smoking [29]. To eliminate the potential impact of cigarette smoking, we compared smoking status and pack-years by SES status. We found that those with higher SES were more likely to be never-smokers and consume fewer cigarettes. Thus, the difference cannot be explained by the smoking difference. More research is needed to clarify the association further.

Previous studies have shown that AL is higher among smokers than never-smokers [2,13,30,31]. Still, there is no study to assess the relationship between smoking-related variables beyond smoking status and AL. In this study, we assessed the impact of smoking status, pack-years of smoking among all study participants, age of smoking initiation, and years of smoking among former and current smokers, respectively, and age of smoking cessation among former smokers. Our results have demonstrated cigarette smoking—not only the amount and duration but also the timing—had a significant influence on AL. As mentioned above, individuals with higher levels of stress are more likely to engage in heavy smoking and are less likely to quit successfully than their counterparts [20,21]. Thus, those who have elevated levels of stress are more susceptible and addicted to smoking behavior, which, consequently, leads to further increased levels of stress. Such action creates a vicious cycle of exacerbating smoking and stress. This is also consistent with our finding that the risk association was only observed among former and never-smokers.

The main limitation of this study is the lack of information on tumor subtypes. So, we don’t know whether the association between adenocarcinoma and squamous carcinoma differs. Also, we only have a one-time measurement of AL, which does not allow us to assess the dynamic changes in AL during the follow-up. Nevertheless, this is the first study to demonstrate the risk association between AL as a biomarker of chronic stress and lung cancer risk in a large epidemiologic study. Further research is warranted to confirm the results of this study.

## 5. Conclusions

In this epidemiologic study, we have reported a significant relationship between higher AL and lung cancer risk. The risk association could be modified by cigarette smoking, demographics, socioeconomics, and other health behaviors. Future studies are needed to further assess the role of AL in lung carcinogenesis. 

## Figures and Tables

**Table 1:** Comparison of characteristics between incident lung cancer cases and cancer-free controls.

Variables	Incident Cases (N = 2517)	Cancer-Free Controls (N = 341,863)	*p* Value
Age at recruitment, mean (SD)	61.6 (5.84)	56.2 (8.10)	<0.001
Years from recruitment to cancer diagnosis, mean (IQR)	7.0 (4.2, 9.4)		
Gender (%)			<0.001
Male	1310 (52.05)	161,113 (47.13)	
Female	1207 (47.95)	180,750 (52.87)	
Race/ethnicity (%)			<0.001
White	2437 (96.82)	322,611 (94.37)	
Black	25 (0.99)	4905 (1.43)	
Asian	26 (1.03)	8041 (2.35)	
Mixed or others	23 (0.91)	5129 (1.50)	
Missing	6 (0.24)	1177 (0.34)	
Family history of lung cancer (%)			<0.001
Yes	350 (13.91)	26,366 (7.71)	
No	915 (36.35)	135,222 (39.55)	
Missing	1252 (49.74)	180,275 (52.73)	
Education (%)			<0.001
High school or less	975 (38.74)	153,541 (44.91)	
College/professional	537 (21.33)	129,149 (37.78)	
Missing	1005 (39.93)	59,173 (17.31)	
Employment status (%)			<0.001
Unemployed	295 (11.72)	28,091 (8.22)	
Employed	887 (35.24)	201,474 (58.93)	
Retired	1309 (52.01)	109,100 (31.91)	
Missing	26 (1.03)	3198 (0.94)	
Income (%)			<0.001
<£31,000	1485 (59.00)	137,853 (40.32)	
≥£31,000	557 (22.13)	155,840 (45.59)	
Missing	475 (18.87)	48,170 (14.09)	
Townsend deprivation score (%)			0.001
Low	915 (36.35)	172,446 (50.44)	
High	1601 (63.61)	169,013 (49.44)	
Missing	1 (0.04)	404 (0.12)	
Smoking (%)			<0.001
Never	336 (13.35)	188,406 (55.11)	
Former	1121 (44.54)	117,153 (34.27)	
Current	1039 (41.28)	35,102 (10.27)	
Missing	21 (0.83)	1202 (0.35)	
Physical activity (%)			0.001
Low	439 (17.44)	52,179 (15.26)	
Moderate	781 (31.03)	112,916 (33.03)	
High	721 (28.65)	112,585 (32.93)	
Missing	576 (22.88)	64,183 (18.77)	
Alcohol consumption (%)			<0.001
Special occasions or never	602 (23.92)	65,223 (19.08)	
Moderate	818 (32.50)	126,285 (36.94)	
Heavy	1093 (43.42)	150,071 (43.90)	
Missing	4 (0.16)	284 (0.08)	
Sleeplessness (%)			<0.001
Never/rarely	495 (19.67)	83,804 (24.51)	
Sometimes	1182 (49.96)	163,086 (47.71)	
Usually	840 (33.37)	94,676 (27.69)	
Missing	0 (0.08)	297 (0.09)	

**Table 2:** AL scores and score categories between lung cancer cases and controls.

	Incident Cases (N = 2517)	Cancer-Free Controls (N = 341,863)	*p* Value
**AL Score**			<0.001
0	65 (2.58)	23,032 (6.74)	
1	245 (9.73)	61,586 (18.01)	
2	401 (15.93)	67,493 (19.74)	
3	569 (22.61)	69,734 (20.40)	
4	540 (21.45)	57,359 (16.78)	
5	411 (16.33)	38,210 (11.18)	
6	206 (8.18)	17,956 (5.25)	
7	70 (2.78)	5317 (1.56)	
8	8 (0.32)	1047 (0.31)	
9	2 (0.08)	123 (0.04)	
10	0	5 (<0.01)	
11	0	1 (<0.01)	
**AL (continuous), Mean (SD)**	3.47 (1.58)	3.35 (1.67)	<0.001
**AL category**			
Low (0~2)	711 (28.25)	152,111 (44.49)	<0.001
High (>2)	1806 (71.75)	189,752 (55.51)	
	**Univariate**	Multivariate *	
	**HR (95% CI)**	**HR (95% CI)**	
**AL, continuous, Per one unit**	1.23 (1.20, 1.26)	1.02 (0.99, 1.04)	
**AL category**			
Low	Reference	Reference	
High	2.08 (1.91, 2.27)	1.15(1.05, 1.25)	

* Adjusted by age, gender, race, family history, employment, education, income, Townsend deprivation index, smoking status, pack-years of smoking, alcohol consumption, physical activity, and sleep quality.

**Table 3:** AL and lung cancer risk stratified by sociodemographic factors and healthy behaviors.

Age group (median)	<57 years old (n = 173,224)			≥57 years old (n = 171,156)		
AL: high (>2) vs. Low (≤2)	1.35 (1.13, 1.62)			1.26 (1.14, 1.40)		
Gender	Male (n = 162,423)			Female (n = 181,957)		
AL: high (>2) vs. Low (≤2)	1.19 (1.04, 1.37)			1.36 (1.11, 1.54)		
Race	White (n = 325,048)			Non-white (n = 18,149)		
AL: high (>2) vs. Low (≤2)	1.23 (1.12, 1.35)			0.74 (0.45, 1.22)		
Family history of cancer	No (n = 136,137)			Yes (n = 26,716)		
AL: high (>2) vs. Low (≤2)	1.14 (0.98, 1.32)			1.00 (0.79, 1.28)		
Education	≤High school (n = 154,516)			>High school (n = 129,686)		
AL: high (>2) vs. Low (≤2)	1.11 (0.97, 1.28)			1.28 (1.06, 1.55)		
Income	<£31,000 (n = 139,338)			≥£31,000 (n = 156,397)		
AL: high (>2) vs. Low (≤2)	1.15 (1.02, 1.29)			1.31 (1.09, 1.58)		
Townsend deprivation score	Low (n = 173,361)			High (n = 170,614)		
AL: high (>2) vs. Low (≤2)	1.26 (1.09, 1.47)			1.18 (1.05, 1.32)		
Employment	Unemployed (n = 28,386)		Employed (n = 202,361)		Retired (n = 110,409)	
AL: high (>2) vs. Low (≤2)	1.10 (0.83, 1.43)		1.23 (1.06, 1.43)		1.25 (1.10, 1.42)	
Smoking status	Never (n = 188,742)		Former (n = 118,274) *		Current (n = 36,141) *	
AL: high (>2) vs. Low (≤2)	0.93 (0.74, 1.17)		1.38 (1.20, 1.59)		1.17 (1.02, 1.35)	
Alcohol consumption	Occasional/never (n = 65,825)		Moderate (n = 127,103)		Heavy (n = 151,164) *	
AL: high (>2) vs. Low (≤2)	1.11 (0.93, 1.34)		1.24 (1.06, 1.46)		1.25 (1.09, 1.44)	
Physical activity	Low (n = 52,618)		Moderate (n = 113,697)		High (n = 113,306)	
AL: high (>2) vs. Low (≤2)	1.06 (0.85, 1.33)		1.27 (1.07, 1.49)		1.19 (1.01, 1.40)	
Sleeplessness	Never/rarely (n = 84,299)		Sometimes (n = 164,268)		Usually (n = 95,516)	
AL: high (>2) vs. Low (≤2)	1.32 (1.07, 1.63)		1.23 (1.07, 1.40)		1.13 (0.97, 1.32)	

Adjusted by age, gender, race, family history, employment, education, income, Townsend deprivation index, smoking status, pack-years of smoking, alcohol consumption, physical activity, and sleep quality, as appropriate. * Significant interaction was observed.

**Table 4:** Comparison of AL score by selected smoking-related variables.

Smoking-Related Variable	Number	AL (Mean/SD)	*p*-Value
Smoking status			<0.001
Never	188,742	2.70 (1.69)	
Former	118,274	3.08 (1.71)	
Current	36,141	3.08 (1.75)	
*p* for trend		<0.001	
Pack-years of smoking			<0.001
Never	188,742	2.71 (1.69)	
Q1 (>0 to ≤9.75)	25,252	2.66 (1.67)	
Q2 (>9.75 to ≤18.75)	25,648	3.00 (1.69)	
Q3 (>18.75 to ≤31.50)	25,796	3.27 (1.69)	
Q4 (>31.50)	25,776	3.75 (1.65)	
*p* for trend		<0.001	
**Among former smokers**			
Age of smoking initiation			<0.001
Q1 (≤15 years old)	23,377	3.41(1.72)	
Q2 (>15 to ≤17 years old)	23,385	3.23(1.69)	
Q3 (>17 to ≤18 years old)	13,004	3.11(1.67)	
Q4 (>18 years old)	18,778	3.07(1.70)	
*p* for trend		<0.001	
Age of smoking cessation			<0.001
Q1 (≤30 years old)	22,129	2.87 (1.68)	
Q2 (>30 to ≤39 years old)	18,792	3.09 (1.72)	
Q3 (>39 to ≤49 years old)	20,023	3.33 (1.69)	
Q4 (>49 years old)	17,676	3.67 (1.61)	
*p* for trend		<0.001	
Years of smoking			<0.001
Q1 (≤13 years)	21,415	2.82 (1.68)	
Q2 (>13 to ≤21 years)	19,188	3.12 (1.70)	
Q3 (>21 to ≤31 years)	19,529	3.34 (1.69)	
Q4 (>31 years)	18,126	3.69 (1.62)	
*p* for trend		<0.001	
**Among current smokers**			
Age of smoking initiation			<0.001
Q1 (≤15 years)	8973	3.29 (1.76)	
Q2 (>15 to ≤16 years)	4399	3.16 (1.75)	
Q3 (>16 to ≤19 years)	6881	3.07 (1.75)	
Q4 (>19 years)	6053	3.02 (1.74)	
*p* for trend		<0.001	
Years of smoking			<0.001
Q1 (≤30 years)	6837	2.62 (1.77)	
Q2 (>30 to ≤37 years)	6386	3.00 (1.76)	
Q3 (>37 to ≤44 years)	6830	3.36 (1.69)	
Q4 (>44 years)	6253	3.65 (1.60)	
*p* for trend		<0.001	

## Data Availability

The data that support the findings of this study are available from the corresponding author upon reasonable request.

## Supplementary Materials

The following supporting information can be downloaded at: https://www.mdpi.com/article/10.3390/cancers16183235/s1, Table S1: Codes used to identify lung cancer cases (Study censoring date: 31 December 2020). Table S2: Distribution and high-risk cutoff points for individual biomarkers of AL scores (N = 456,263). Figure S1: Flow chart of the study population.
