# Supplementary material for: Allostatic Load, Cigarette Smoking, and Lung Cancer Risk
Source: Cancers (Basel). 2024 Sep 23;16(18):3235. doi: 10.3390/cancers16183235 (PMC11429585; doi:10.3390/cancers16183235)

Supplemental Tables:

Table S1: Codes used to identify lung cancer cases (Study censoring date:12/31/2020)

Categories	Frequency (%)	ICD10 codes
**Lung cancer cases**		
• Prevalent:	269 (0.05%)	Codes start with C34
• Incident:	3646(0.73%) [208(Incident >1 year); 3438(Incident <1 year))]	
**Other cancers(except for non-melanoma skin cancer)**		
• Prevalent:	22070(4.39%)	Codes start with C except codes for PC and non-melanoma skin cancer
• Incident:	43425(8.65%)	
**In situ carcinoma**		
• Prevalent:	6582(1.31%)	Codes start with D0-D09
• Incident:	5748(1.14%)	
**Neoplasm of unknown nature or behavior**		
• Prevalent:	1215(0.24%)	Codes start with D37- D48
• Incident:	1878(0.37%)	
**Benign neoplasms**		
• Prevalent:	655(0.13%)	Codes start with D10-D36
• Incident:	685(0.14%)	
**non-melanoma skin cancer**		
• Prevalent:	8702(1.73%)	Codes start with C44
• Incident:	22505(4.48%)	
**Unknow prevalent cancers**	6277(1.25%)	Subject only with cancer diagnosed time, no Codes
**Non-case controls**	378584 (75.38%)	Remaining codes or subjects with no code assigned
**Total**	502241 (100%)	

- Subject with any cancer developed before enrolment was excluded.
- Subject with any cancer other than lung cancer during follow-up were censored at the date of diagnosis.

Table S2: Distribution and high-risk cutoff points for individual biomarkers of AL scores (N=456263)

	Cutoff Value	Mean(SD)	N (%) at Risk	N (%)missing
Waist to hip ratio	>=0.9 for male or >=0.85 for female	0.87(0.09)	226026(49.54%)	2051(0.45%)
Pulse rate	>100	69.26(11.84)	4589(1.01%)	27372(6.00%)
SBP (mm Hg)	>=140	137.70(18.62)	181106(39.69%)	27376(6.00%)
DBP (mm Hg)	>=90	82.26(10.16)	96277(21.10%)	27372(6.00%)
HDL (mmol/L)	>1 for male or >1.3 for female	1.44(0.38)	38681(8.48%)	65422(14.34%)
LDL (mmol/L)	>3.4	3.55(0.87)	283925(62.23%)	29659(6.50%)
Total Cholesterol(mmol/L)	>5.2	5.69(1.14)	279960(61.36%)	29428(6.45%)
Triglycerides(mmol/L)	>=1.7	1.75(1.03)	170781(37.43%)	29767(6.52%)
C-reactive protein(mg/L)	>3	2.56(4.27)	95115(20.85%)	30336(6.65%)
Creatinine(umol/L)	>114.9 for male or >97.2 for female	72.36(18.14)	6033(1.32%)	29638(6.50%)
Glycated hemoglobin (HbA1c)mmol/mol	>48	36.10(6.80)	15826(3.47%)	32332(7.09%)
Medication history	Yes		123443(27.06%)	7926(1.74%)

Supplement Figure S1: Flow chart of the study population

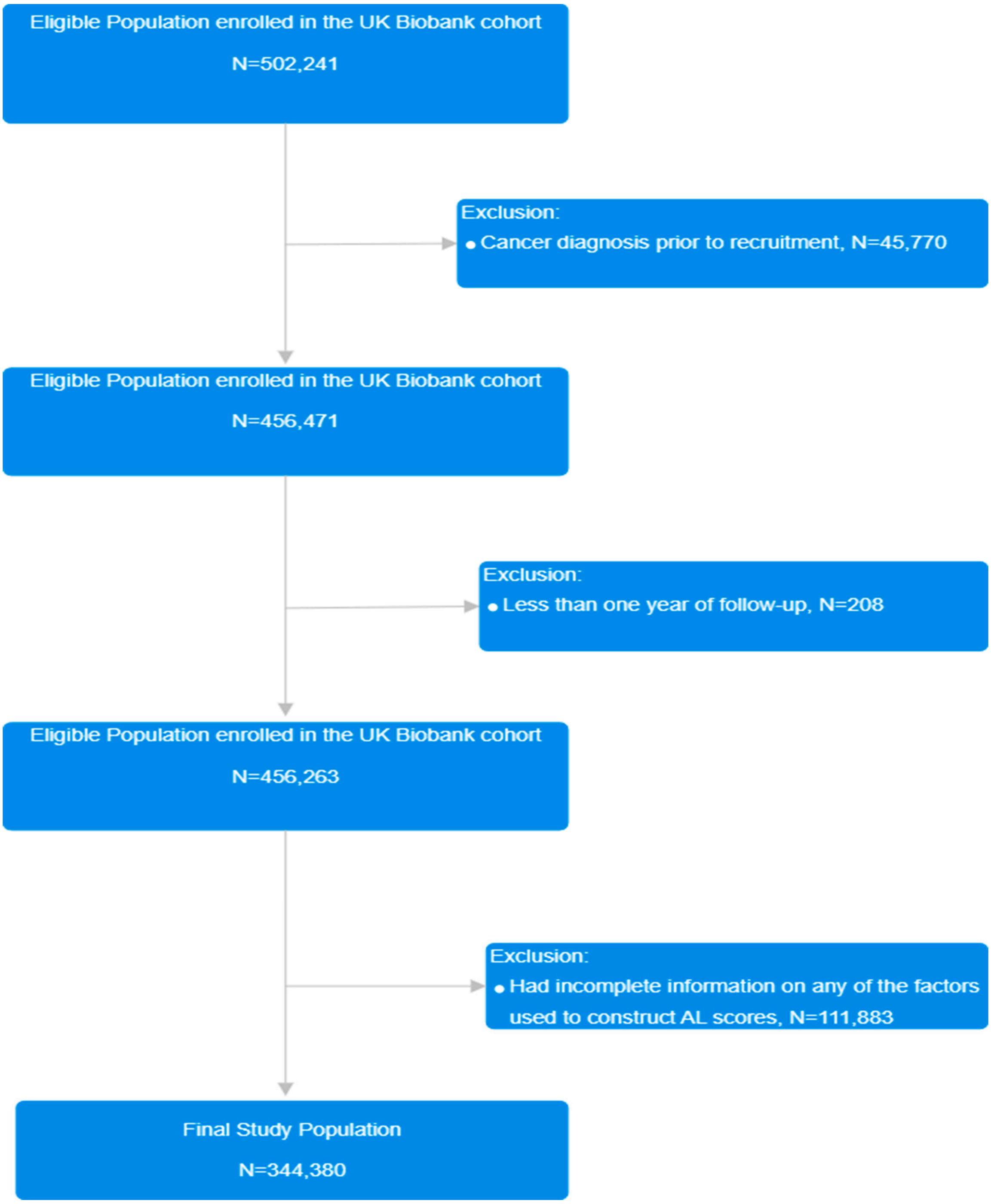

Supplement: Supplementary file 1 [file cancers-16-03235-s001.zip › cancers-3220098-supplementary.pdf]
